# Left atrial remodeling in mitral regurgitation: A combined experimental-computational study

**DOI:** 10.1371/journal.pone.0271588

**Published:** 2022-07-15

**Authors:** Sjoerd Bouwmeester, Tim van Loon, Meike Ploeg, Thomas P. Mast, Nienke J. Verzaal, Lars B. van Middendorp, Marc Strik, Frans A. van Nieuwenhoven, Lukas R. Dekker, Frits W. Prinzen, Joost Lumens, Patrick Houthuizen

**Affiliations:** 1 Department of Cardiology, Catharina Hospital Eindhoven, Eindhoven, The Netherlands; 2 Department of Biomedical Engineering, CARIM School for Cardiovascular Diseases, Maastricht University, Maastricht, The Netherlands; 3 Department of Physiology, CARIM School for Cardiovascular Diseases, Maastricht University, Maastricht, The Netherlands; 4 Bordeaux University Hospital (CHU), Cardio-Thoracic Unit, Pessac, France; 5 Department of Biomedical Technology, Eindhoven University of Technology, Eindhoven, The Netherlands; Universitatsklinikum Wurzburg, GERMANY

## Abstract

**Aims:**

Progressive changes to left atrial (LA) structure and function following mitral regurgitation (MR) remain incompletely understood. This study aimed to demonstrate potential underlying mechanisms using experimental canine models and computer simulations.

**Methods:**

A canine model of MR was created by cauterization of mitral chordae followed by radiofrequency ablation-induced left bundle-branch block (LBBB) after 4 weeks (MR-LBBB group). Animals with LBBB alone served as control. Echocardiography was performed at baseline, acutely after MR induction, and at 4 and 20 weeks, and correlated with histology and computer simulations.

**Results:**

Acute MR augmented LA reservoir and contractile strain (40±4 to 53±6% and -11±5 to -22±9% respectively, p<0.05). LA fractional area change increased significantly (47±4 to 56±4%, p<0.05) while LA end-systolic area remained unchanged (7.2±1.1 versus 7.9±1.1 cm^2^ respectively, p = 0.08). LA strain ‘pseudonormalized’ after 4 weeks and decompensated at 20 weeks with both strains decreasing to 25±6% and -3±2% respectively (p<0.05) together with a progressive increase in LA end-systolic area (7.2±1.1 to 14.0±6.3 cm^2^, p<0.05). In the LBBB-group, LA remodeling was less pronounced. Histology showed a trend towards increased interstitial fibrosis in the LA of the MR-LBBB group. Computer simulations indicated that the progressive changes in LA structure and function are a combination of progressive eccentric remodeling and fibrosis.

**Conclusion:**

MR augmented LA strain acutely to supranormal values without significant LA dilation. However, over time, LA strain gradually decreases (pseudornormal and decompensated) with LA dilation. Histology and computer simulations indicated a correlation to a varying degree of LA eccentric remodeling and fibrosis.

## Introduction

Mitral regurgitation (MR) causes volume overload of both the left ventricle (LV) and atrium (LA). Although guidelines focus on the impact on LV function to indicate intervention, the LA is the first chamber to receive the volume excess [1]. Not surprisingly, LA dilation, as a marker of LA remodeling, is associated with increased cardiovascular morbidity and mortality irrespective of LV function [2,3].

Traditionally, standard echocardiographic (LA volume) and Doppler parameters (transmitral and pulmonary vein flow velocities) are used as indirect measures to assess the effects of MR on LA structure and function [4]. Speckle-tracking echocardiography, however, allows more direct assessment of LA function by myocardial deformation (strain) and may better reflect functional LA changes due to MR [5]. Little is known about the chronological evolution of LA strain from the onset of an acute MR to the chronic phase and what underlying mechanisms are responsible for this evolution.

The aim of this study was to provide new insight into the mechanisms of LA structural and functional remodeling after acute MR. The study made use of the same canine models as previously published [6], whose original aim was to investigate the electromechanical effects of LBBB. Two models were used: 1) radiofrequency ablation-induced left bundle-branch block (LBBB group); and 2) severe MR creation followed by LBB ablation after 4 weeks (MR-LBBB group). Changes in LA structure and function were assessed by echocardiography. Furthermore, histology and gene expression analysis were performed, as well as computer simulations to identify potential underlying myocardial disease mechanisms.

## Material and methods

All animal procedures complied with the Dutch Law on Animal Experimentation and the European Directive for the Protection of Vertebrate Animals used for Experimental and Other Scientific Purposes (86/609/EU). The Experimental Animal Committee of Maastricht University approved the study. Dogs were purchased from a commercial party approved by the Dutch Veterinary Inspection.

### Study design

**Fig 1:**
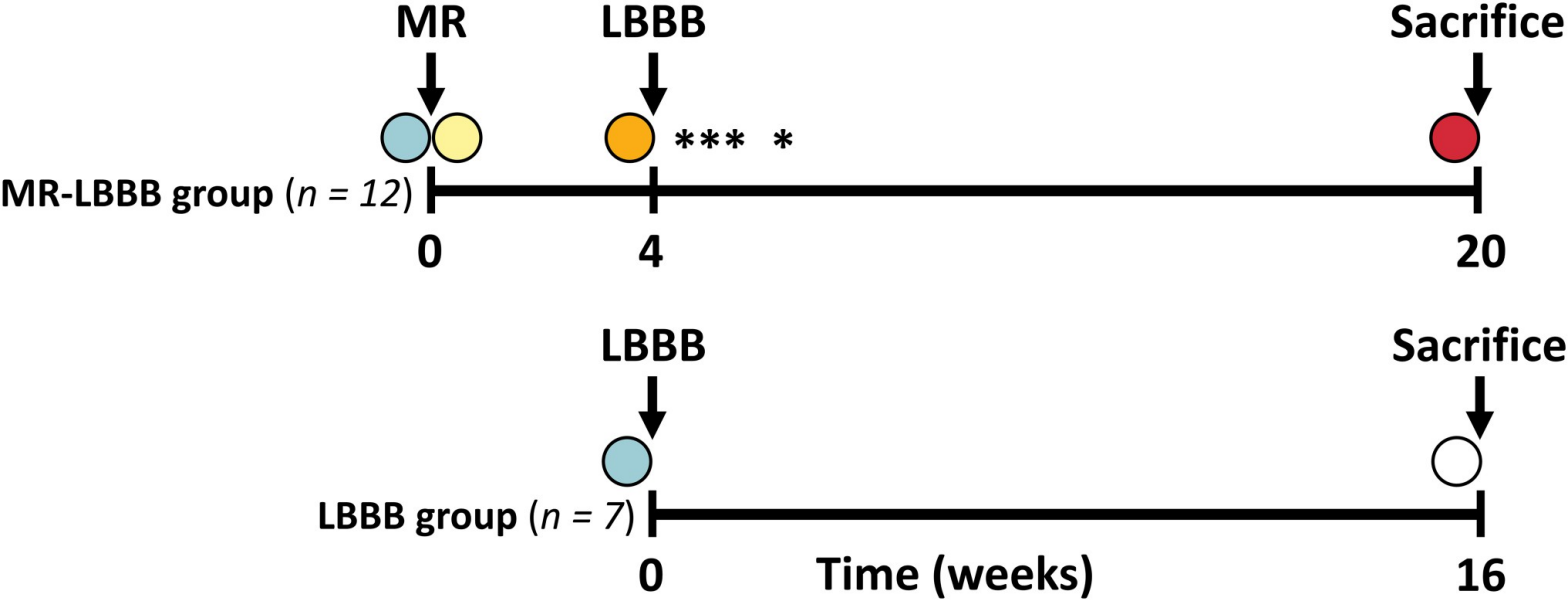
Timeline schematic of the canine experiments. MR-LBBB model: Creation of mitral regurgitation after baseline followed by LBBB induction after 4 weeks. LBBB model: LBBB induction after baseline. Circles indicate the time points of echocardiographic and hemodynamic measurements at baseline (blue), acute MR (yellow), 4 weeks (yellow), 16 weeks (white) and 20 weeks (red). The asterisks in the MR-LBBB group indicate death due to heart failure, all within 6 weeks. MR = mitral regurgitation; LBBB = left bundle branch block.

All experiments were performed under general anesthesia with continuous infusion of midazolam (0.25 mg/kg/h) and sufentanil (3 μg/kg/h) after induction with thiopental (300 mg). Acute severe MR was induced in 12 adult mongrel dogs using a customized electrophysiology catheter with a hook at the distal tip, which was inserted into the carotid artery and advanced into the LV. After grasping one or more chordae, the hook was cauterized to burn through the chordae. This process was repeated until fluoroscopic and echocardiographic guidance indicated severe MR using the integrative approach according to the European Association for Cardiovascular Imaging (EACVI) recommendations [7]. As the original study aimed to investigate electromechanical effects of LBBB, the dogs underwent radiofrequency ablation of the left bundle branch 4 weeks later (MR-LBBB group). During the procedures and at the final day of the protocol, standardized echocardiographic measurements were performed. To distinguish between the effects of MR and LBBB, a second group of 7 dogs with only LBBB (LBBB group) was used as control group (Fig 1). Dogs were sacrificed by overdose of anesthetics 16 weeks after induction of LBBB, and atrial tissue was harvested for histology and gene expression analysis.

### Echocardiography

**Fig 2:**
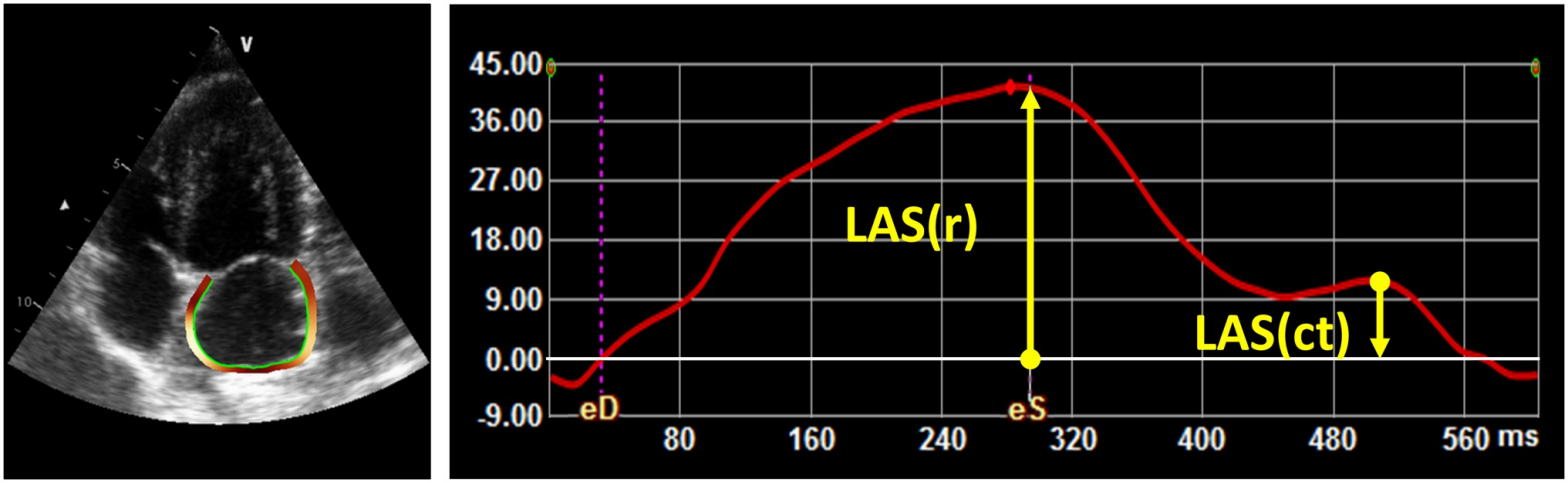
Example of LA strain assessment by 2D speckle-tracking echocardiography at baseline. Four-chamber longitudinal left atrium strain (LAS). Arrows represent reservoir (r) and contractile (ct) phase (42% and -12%, respectively).

Echocardiography was performed in the MR-LBBB group at baseline, directly after induction of MR, and after 4 and 20 weeks. LBBB dogs only underwent echocardiography at baseline and 16 weeks. A Vivid 7 system (GE Vingmed, Horten, Norway) with a 2.5 MHz probe was used with commercially available software to analyze conventional echocardiography parameters (EchoPAC 3.0, GE Vingmed Ultrasound, Horten, Norway). LV end-systolic, end-diastolic volume, and ejection fraction (LVESV, LVEDV, LVEF) were measured on a apical 4-chamber view by modified single-plane Simpson’s rule. LA end-systolic area and LA end-diastolic area expressed in cm^2^ were calculated by tracing the LA endocardium in the apical 4-chamber. LA fractional area change (LAFAC) was calculated with the formula: LAFAC (%) = 100% * [(LA end-systolic area–LA end-diastolic area)/LA end-systolic area] [8]. Commercially available software (TOMTEC ARENA, 2D CPA 1.3, TOMTEC Imaging Systems GmbH, Unterschleißheim, Germany) was used to measure LA strain. As recommended by the Taskforce [9], LA strain was measured on a non-foreshortened apical four-chamber view. The LA border was semi-automatically drawn after a 3-point clicking method followed by manual adjustment if required. The zero-strain reference was defined at LV end-diastole (i.e. mitral valve closure). LA reservoir strain (LAS(r)) was measured as difference of the strain value at mitral valve opening minus LV end-diastole. LA contractile strain (LAS(ct)) was calculated as difference of LAS(r) minus the strain value at onset of atrial contraction. Fig 2 shows an example of the echocardiographic assessment of LA strain.

### Histology and gene expression analysis

Cryosections of 7 μm were cut from the LA of each dog at − 22°C. Antigen fixation was performed by submerging slides in ice-cold acetone for 10 min. The degree of fibrosis was determined in tissue sections stained with 0.1% Sirius Red. The percentage of collagen was determined using ImageJ in 8 separate pictures (200x magnification, Leica Microscope) per atrial section.

Total RNA was isolated from atrial tissue using an RNA isolation kit (Omega Biotek, Norcross, GA, USA) and reversed transcribed into cDNA using the iScript cDNA synthesis kit (Biorad, Hercules, CA, USA) according to the manufacturer’s instructions. Real-time PCR was performed on CFX96 Touch real-time PCR detection system using iQ SYBR-Green Supermix (Biorad). Gene expression level of collagen type 1 (Col1a1) was normalized using the housekeeping gene cyclophilin-A (Cyclo), and their relative expression was calculated using the comparative threshold cycle (Ct) method by calculating 2^ΔCt^ (e.g., 2^(Cyclophilin Ct–Col1a1 Ct)^).

### Computer model simulations

Computer simulations of LA and LV myocardial deformation were performed to identify potential underlying pathophysiological substrates underlying the echocardiographic and histological findings in the canine experiments. In addition, simulations allow for the differentiation between the effects of MR and LBBB to LA function, by simulating these pathologies in isolation.

The open source CircAdapt model of the human heart and circulation was used (www.circadapt.org) [10], as previous studies have demonstrated the potential of this model to simulate cardiovascular mechanics and hemodynamics during valve regurgitation [11,12], dyssynchrony [13], and the combination of both [14]. A reference simulation representing a healthy cardiovascular system under baseline conditions was used as a starting point (see **Supplemental Material** for a detailed description of model initialization). Acute MR was simulated by increasing mitral effective regurgitant orifice area (EROA) from 0 to 0.40 cm^2^. To represent the compromised hemodynamics status after MR, systemic flow and mean arterial pressure were reduced from 5.1 to 3.6 L/min and 92 to 75 mmHg, respectively, as previously published [11]. These changes led to a severe MR as characterized by a mitral regurgitant fraction (RF) >50%. All other model parameters were left unchanged compared to the reference simulation.

LA eccentric hypertrophy and interstitial fibrosis formation are known structural changes caused by MR [15]. To differentiate the possible effects of both LA (patho-)physiological alterations on echocardiographic parameters, two LA myocardial tissue substrates of various severities were simulated (see **Supplemental Material** for a detailed description of the simulation methodology):

1. **LA eccentric hypertrophy**: gradual simultaneous increase of LA wall mass and area from 100 (normal LA geometry) to 300% in 10% increments;
2. **LA passive stiffness**: gradual increase of LA myocardial stiffness from 100 (normal compliance) to 600% in 10% increments.

In each simulation, the change in LAA was compared to the reference simulation, and the strain indices LAS(r) and LAS(ct) were quantified, with zero-strain reference set at mitral valve closure.

To demonstrate the contribution of LBBB in the canine MR-LBBB group, we repeated all the above-mentioned simulations with LBBB. As previously published [13], LBBB was simulated by delaying the mean LV free wall activation time from 0 (synchronous activation) to 30 milliseconds (mild electromechanical substrate), and 60 milliseconds (severe electromechanical substrate), relative to the mean septal- and right ventricular free wall activation times. In a previous study, LBBB has been reported to induce LV eccentric hypertrophy [16]. Hence, analogous to simulating LA eccentric hypertrophy, LV wall mass and area were simultaneously increased to 110% and 120% in the mild and severe electromechanical substrate, respectively. All simulations were performed using MATLAB (R2019a, Mathworks, Natick, MA).

### Statistical analysis

Continuous variables are expressed as median (interquartile range) or mean (standard deviations) when appropriate. Normality of distribution was assessed with the Kolmogorov-Smirnov test. The paired sample Wilcoxon rank-sum test was used to compare hemodynamic and echocardiographic differences between different time points. Continuous variables between MR-LBBB and LBBB groups were compared with the Student’s t test or Mann-Whitney U test as appropriate. Statistical analyses were performed using SPSS version 25 (IBM Corporation, Armonk, NY). Statistical significance was assigned at p<0.05.

## Results

All procedures were successfully performed in the MR-LBBB (n = 12) and LBBB (n = 7) group, however 4 MR-LBBB animals died because of severe heart failure (Fig 1), all within 2 weeks after LBBB induction on top of the MR. The LBBB group had no premature deaths nor development of MR. Baseline data were comparable for both groups (Table 1).

**Table 1:** Overview of echocardiographic and hemodynamic measurements at baseline, acute, subacute, and chronic phase.

	MR-LBBB				LBBB	
	n = 12			n = 8	n = 7	
Phase:	Baseline	Acute-MR	4 weeks	20 weeks	Baseline	16 weeks
Heart rate–bpm	82 ± 26	101 ± 37	86 ± 25	73 ± 24	75 ± 40	79 ± 38
**Echocardiographic LA parameters**						
LA end-systolic area—cm^2^	7.4 [6.3–7.8]	8.0 [7.1–8.9]	13 [8.6–15.8]^†^	11.6 [10.1–20.7]^†^	7.9 [5.4–9.2]	8.5 [7.0–8.6]
LAFAC—%	47 [44–50]	55 [53–59]^†^	41 [39–44]^†^	33 [25–42]^†^	46 [45–48]	43 [42–46]
LAS(r)—%	40 [37–42]	51 [48–56]^†^	32 [30–35]^†^	29 [18–29]^†^	42 [37–43]	39 [36–40]^†^
LAS(ct)—%	-10 [-8; -12]	-21 [-21; -25]^†^	-7 [-2; -10]^†^	-2 [-1;5]^†^	-8 [-7; -12]	-7 [-4; -8]^†^
**Echocardiographic LV parameters**						
LVEDV—ml	42 [34–45]	53 [42–57]^†^	65 [39–75]^†^	82 [50–106]^†^	44 [39–45]	47 [30–51]
LVEF—%	54 [50–60]	61 [61–69]^†^	64 [59–67]^†^	55 [47–57]	50 [48–52]	34 [24–49]^†^

Data are given as mean ± standard deviation.

^†^ p <0.05 compared to baseline.

LA = left atrial; LAFAC = left atrial fractional area change; LAS(r) = left atrial reservoir strain; LAS(ct) = left atrial contractile strain; LVEDV = left ventricular end-diastolic volume; LVEF = left ventricular ejection fraction; [IQR] = interquartile range.

### Time course of LA dilation and function

**Fig 3:**
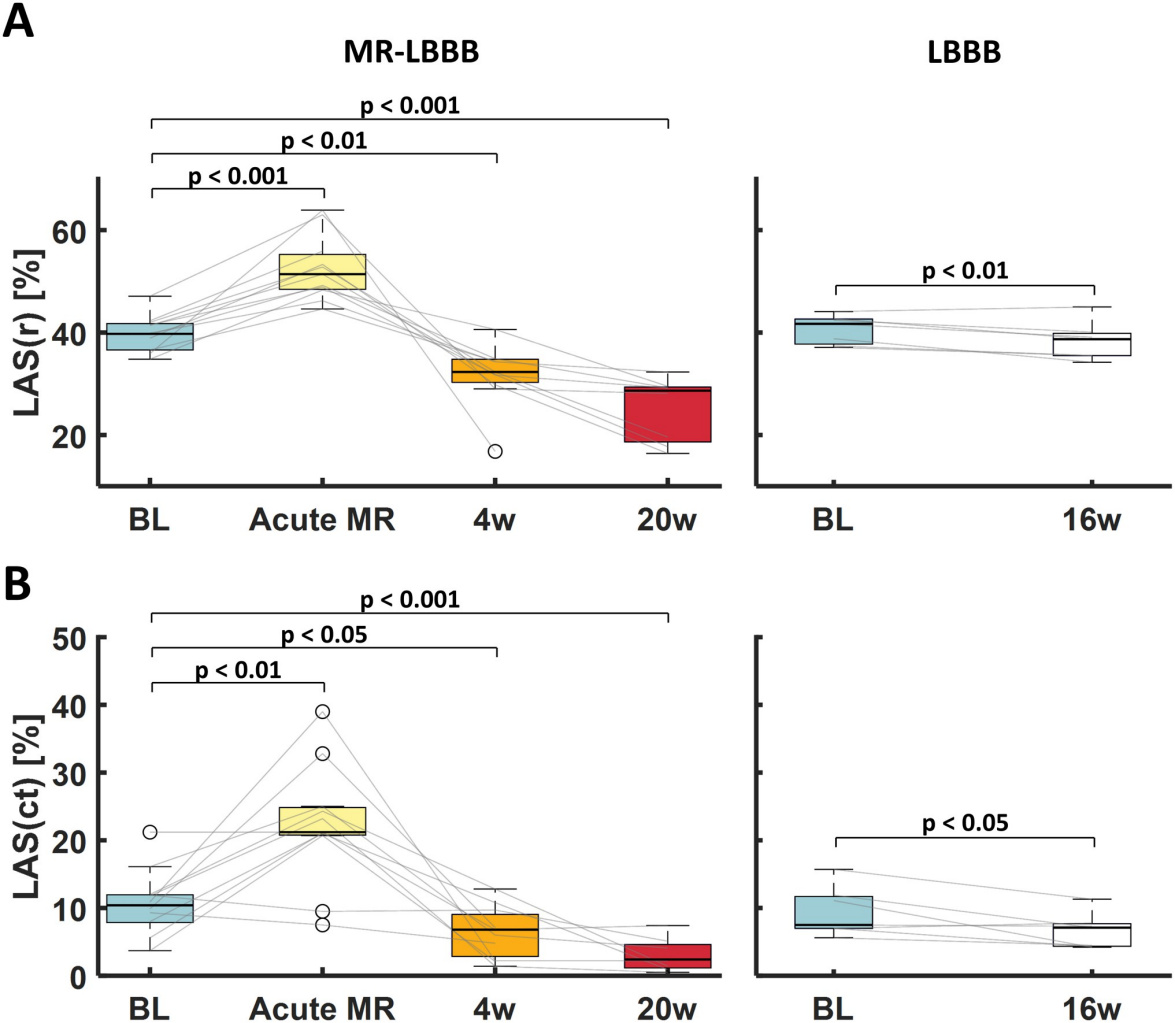
Chronological changes of left atrial strain. **(A-B)** Time course of LA reservoir (LAS(r)) and contractile (LAS(ct)) strain for each group (presented as **boxplots and lines** for individual animals). Statistical significance was assigned at p < 0.05. BL = baseline; LAS(r) = left atrial reservoir strain; LAS(ct) = left atrial contractile strain; LBBB = left bundle branch block, MR = mitral regurgitation; w = weeks.

Table 1 summarizes the time course of all echocardiographic and hemodynamic LA and LV parameters. In MR-LBBB dogs, no significant LA dilation (as quantified by LA end-systolic area) was present directly after MR induction with respect to baseline (7.4 [6.3–7.8] cm^2^ versus 8.0 [7.1–8.9] cm^2^, respectively). However, progressive LA dilation was observed in weeks 4 to 20 post-MR (13 [8.6–15.8] cm^2^ and 11.6 [10.1–20.7] cm^2^, respectively). Moreover, LAFAC increased significantly from 47 [44–50]% at baseline to 55 [53–59]% immediately after MR was induced, and decreased progressively to 41 [39–44]% at week 4 and 33 [25–42]% at week 20. The combination of progressive LA dilation with decreasing LAFAC suggests LA functional deterioration. This is corroborated by the observation and shown in Fig 3 **(left panels)**, in which both LAS(r) and LAS(ct) augmented to supranormal values acutely after MR was induced (40 [37–42] to 51 [48–56]% and -10 [-8;-12] to -21 [-21;-25]%, respectively), whereas in weeks 4 to 20 a significant decline in both strain indices was observed (32 [30–35] to 29 [18–29] % and -7 [-2; -10] to -2 [-1; -5]%, respectively).

No significant LA dilation was present in the LBBB-group, while LAFAC, LAS(r), and LAS(ct) did decrease over time (Fig 3, **right panels**). The MR-LBBB group, however, showed significantly larger decreases in LAFAC, LAS(r), and LAS(ct) compared to the LBBB-group.

### Time course of LV dilation and function

Significant LV dilation and increased (hyperdynamic) LVEF was observed after MR induction in the MR-LBBB group compared to baseline (42 [34–45] to 53 [42–57] ml and 54 [50–60] to 61 [61–69]%, respectively). At 4 weeks, progressive LV dilation (65 [39–75] ml) was accompanied by an insignificant increase in LVEF (64 [59–67]%) followed by further LV dilation (82 [50–106] ml), and accompanied by a LVEF pseudo-normalization at 20 weeks (55 [47–57]%). In the LBBB group a significant decrease in LVEF without LV dilation was observed at 16 weeks compared to baseline (50 [48–52] to 34 [24–49]% and 44 [39–45] to 47 [30–51] ml).

### Histology and gene expression analysis

**Fig 4:**
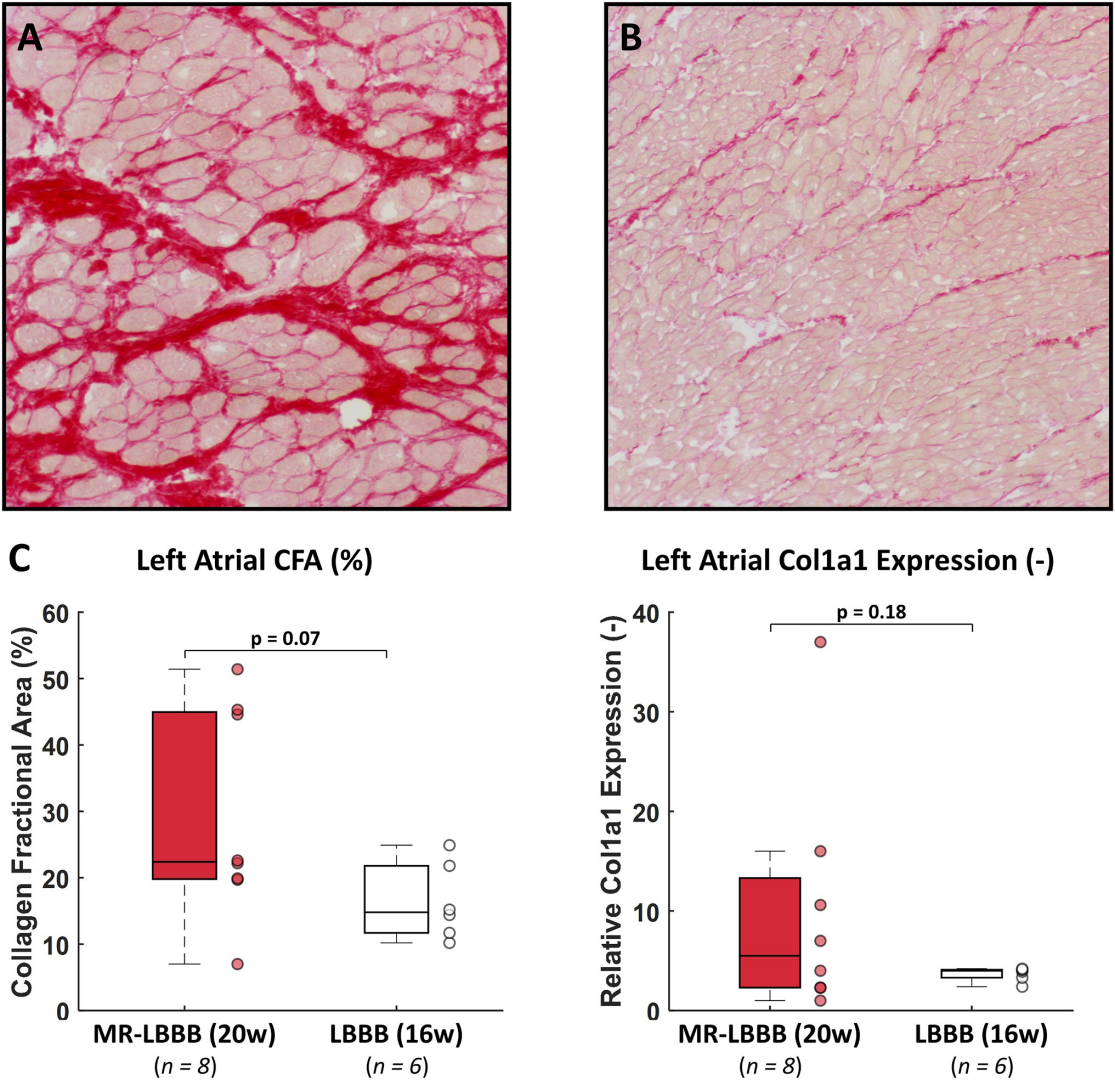
Histological and gene expression analysis of LA tissue. Representative examples of 0.1% Sirius Red stained LA tissue sections of the **(A)** MR-LBBB group and **(B)** LBBB group at 200x magnification. **(C)** Comparison of collagen fractional area (CFA) and relative collagen type 1 (Col1a1) expression to Cyclophilin-A for each group (presented as **boxplots and scatters** for individual animals). A trend towards more interstitial fibrosis and increased Col1a1 expression was found in the MR-LBBB group at 20 weeks, as compared to LBBB group at 16 weeks.

Compared with the LBBB group, LA tissue from the MR-LBBB group showed a trend towards increased interstitial LA fibrosis and Col1A1 expression (Fig 4A to 4B), providing histological and gene expression evidence of LA structural remodeling in this group.

### Computer simulations

**Fig 5:**
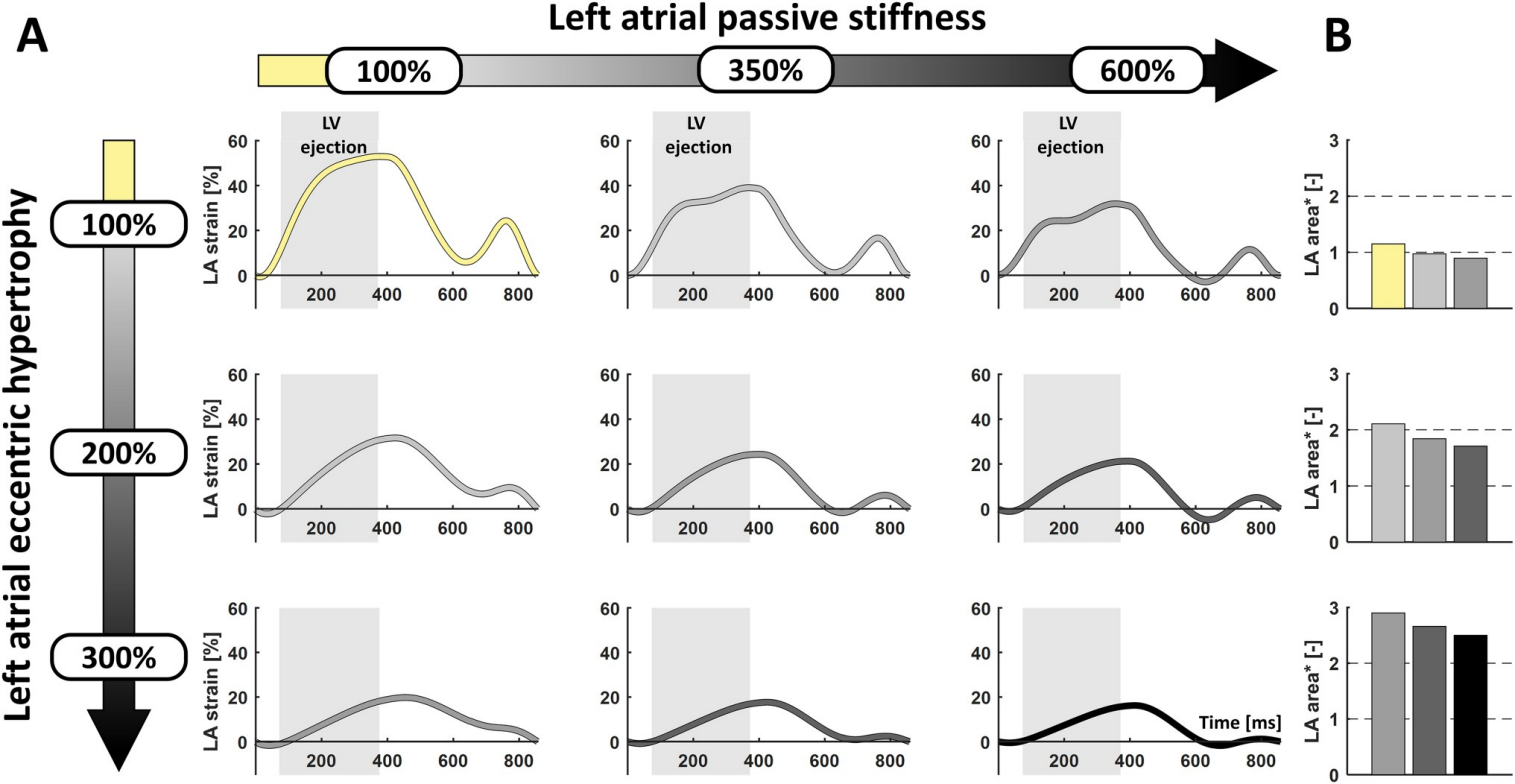
Simulations of LA strain and dilation after acute MR. Simulated changes in **(A)** LA strain and **(B)** LA area (i.e. change in LA end-systolic area relative to baseline simulation) of varying severity of LA passive stiffness (**left-to-right**) and LA eccentric hypertrophy (**top-to-bottom**) starting from the simulation of the acute severe MR phase (**yellow**). Vertical gray areas in the strain panels indicate the period of LV ejection.

In the CircAdapt model, acute induction of severe MR alone hardly affected LA end-systolic area compared to baseline simulation, while LAFAC, LAS(r) and LAS(ct) increased similarly as observed in the MR-LBBB dogs (Table 2). Simulation of structural LA myocardial remodeling in terms of eccentric remodeling and stiffening in the acute MR simulation resulted in marked changes in strain pattern and LA end-systolic area (Fig 5):

1. an increase in **LA eccentric hypertrophy** resulted in *increased* LA end-systolic area together with *decreased* LAS(r) and LAS(ct);
2. a higher **LA passive stiffness** led to a *decreased* LA end-systolic area together with *decreased* LAS(r) and LAS(ct).

**Table 2:** Computer simulation characteristics and derived functional measurements.

	Computer Simulations					
Phase:	Baseline	Acute-MR	4 weeks	20 weeks		
				Synchronous	+30 ms delay	+60 ms delay
**Global hemodynamics**						
Heart rate–bpm	70	70	70	70	70	70
Systemic flow–L/min	5.1	3.6	3.6	3.6	3.6	3.6
Mean arterial pressure–mmHg	92	75	75	75	75	75
**Mitral valve characteristics**						
EROA–cm^2^	0	0.40	0.40	0.40	0.40	0.40
Regurgitant fraction–%	0	52	52	52	54	56
**LA tissue characteristics**						
LA eccentric hypertrophy–%	100	100	180	250	250	250
LA passive stiffness–%	100	100	220	530	530	530
**LV tissue characteristics**						
LV free wall activation delay–ms	0	0	0	0	30	60
LV eccentric hypertrophy–%	100	100	100	100	113	125
**Derived LA functional indices**						
LA end-systolic area* –	1.00	1.15	1.72	2.00	2.00	2.00
LAFAC–%	61	69	55	45	49	52
LAS(r)–%	41	54	31	19	19	22
LAS(ct)–%	14	26	10	3	0	0
**Derived LV functional indices**						
LVEDV–ml	130	150	150	150	175	200
LVEF–%	55	72	72	72	65	58

It was observed that none of the LA tissue parameters alone were sufficient to reproduce changes in LA dilation and function as observed in the MR-LBBB-group. Hence, all simulation combinations were considered and two ‘best-match’ simulations were obtained with LAS(r), LAS(ct), and LA end-systolic area being closest to the average values measured at the 4 and 20 weeks phase. More details on simulation selection are in the **Supplemental Material**.

In-vivo observation at 4 weeks were best reproduced by increasing LA eccentric hypertrophy to 190% and passive stiffness to 225% of normal values (Table 2). The 20 weeks phase was obtained by a further increase in LA eccentric hypertrophy to 260% and passive stiffness to 525%. A more detailed examination on the sensitivity of the LA structural and functional indices with respect to different degrees of LA eccentric remodeling and passive stiffness is available in the **Supplemental Material**.

**Fig 6:**
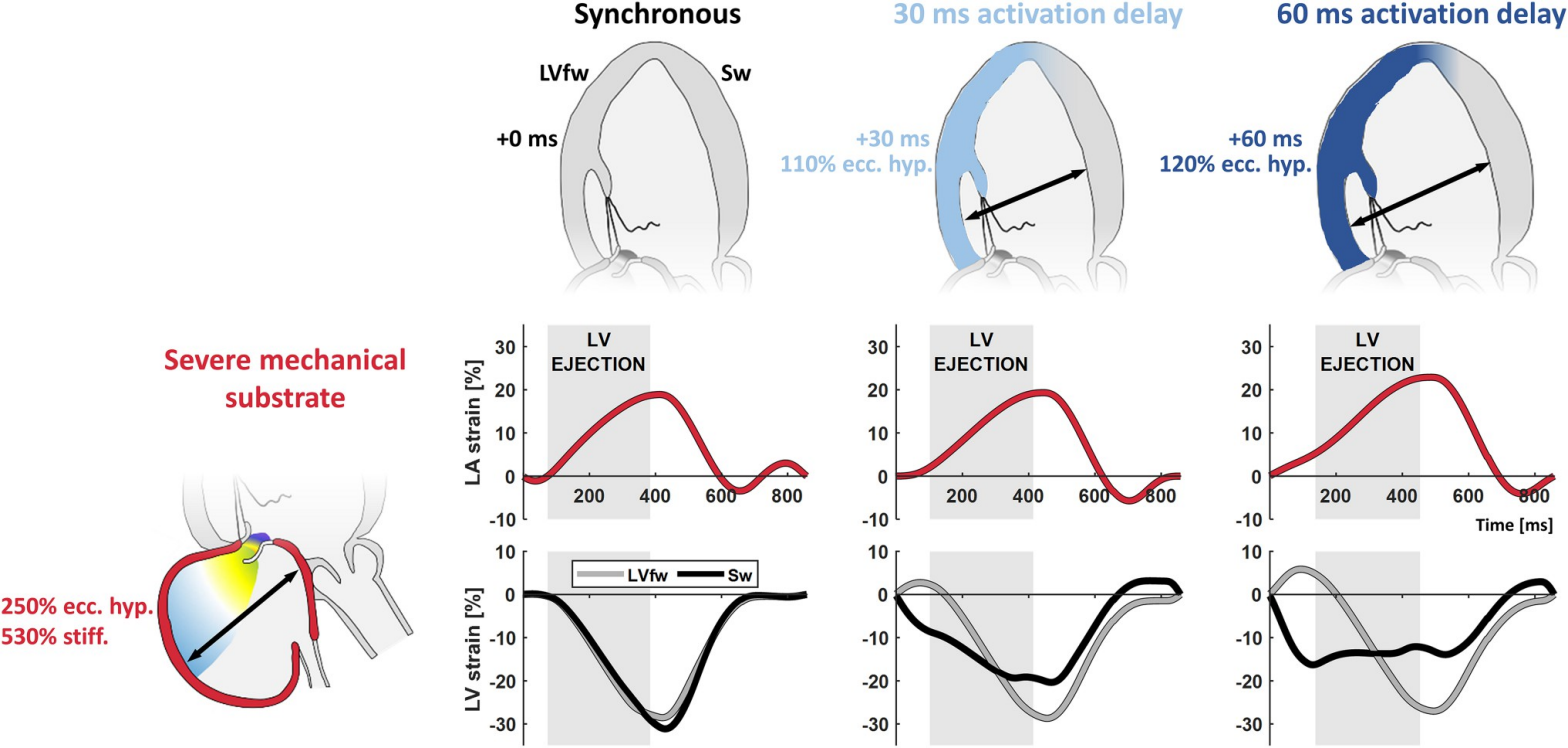
Simulations of LA and LV strain with MR after induction of LBBB. Effect of increasing LV electromechanical activation delay in addition to acute MR. Electromechanical activation delay is characterized by a delayed onset of LV filling, increased LAS(r) and decreased LAS(ct). Vertical gray areas in the strain panels indicate the period of LV ejection. LVfw = left ventricular free wall; Sw = septal wall; ecc. hyp. = eccentric hypertrophy; stiff. = passive stiffness.

None of the abovementioned changes to the LA myocardium led to a change in LV dilation and function (Table 2). However, adding LBBB to the simulations with severe LA mechanical dysfunction (congruent with the 20 weeks in-vivo observations) did impair LV function, characterized by LV dilation (i.e. LVEDV), decreased LVEF (Table 2), and heterogeneous longitudinal LV strain (Fig 6).

Concerning the interaction with LBBB, a delayed onset of LV filling was observed with a shorter LV diastolic duration. As a result, LBBB acutely increased LAS(r) and decreased LAS(ct).

Overview of computer simulation characteristics, the underlying LA myocardial tissue substrate, and derived echocardiographic measurements of the simulated baseline, acute, 4 and 20 weeks phase. *LA end-systolic area is calculated relative to baseline simulation. EROA = effective regurgitant orifice area; LAFAC = left atrial fractional area change; LAS(ct) = left atrial contractile strain; LAS(r) = left atrial reservoir strain; LVEDV = left ventricular end-diastolic volume; LVEF = left ventricular ejection fraction; MR = mitral regurgitation.

## Discussion

The present study provides insight how LA volume and function change after induction of acute MR in a canine model. We demonstrated that acute MR does not lead to LA dilation in the acute phase, but does increase both LA reservoir and contractile strain function. At later stages, there is progressive LA dilation with decrease in LA reservoir and contractile function (**Central Illustration**). Both histology and computer simulations support the hypothesis that these changes of LA mechanical function are likely caused by a combination of eccentric hypertrophy and fibrosis. This study also illustrates that LA dysfunction precedes LV failure.

### MR acutely augments LA function

In a clinical situation of chronic mitral regurgitation, the volume-overloaded LA has to accommodate the excess in regurgitant volume by dilation in order to protect the pulmonary vasculature from high filling pressures [17,18]. As LA compliance is limited in acute MR, filling pressures increase and can result in pulmonary edema. In the acute phase of our experiment, there is an instant increase in LA stroke volume together with augmentation of both reservoir and contractile function. Given the absence of LA dilation, the increased strain can only be explained by a small (albeit not-significant) increase in LA end-systolic volume with related stretch-induced increase of LA myocardial contractility (i.e. Frank-Starling length-tension relationship) [19]. This in turn augments the LA booster pump and increases the LAS(ct). Indeed, computer simulation of acute MR confirm this hypothesis of enhanced LA contractile function as LA strain increases without significant LA dilation, while all model parameters governed with myocardial tissue behavior are kept constant. Hence, the improved contractility leads to an acute rise in LA stroke volume so that the healthy atrial myocardium is able to compensate for the excess regurgitant volume.

### LA decompensation following prolonged volume overload

In later stages, the reduction in LAS(r) and LAS(ct) together with progressive LA dilation indicate that the LA myocardium cannot cope with the chronic volume overload caused by MR. Simulations have shown that this progressive LA failure, cannot be explained by LA dilation alone, as both eccentric hypertrophy and fibrosis were needed to reproduce the in-vivo LA volume and strain observations (Fig 5).

It is known that the LA responds to the excess volume load with a range of adaptive and maladaptive processes. These include myocyte growth, hypertrophy, and finally, apoptosis and necrosis [5,15]. Together with excessive fibroblast proliferation, myolysis is enhanced and, consequently, the contractile apparatus is affected [20]. These findings correspond to the progressive increase in LA eccentric hypertrophy and fibrosis as shown by the computer simulations and by the histological examination.

Our observation that LA strain progresses from supranormal (acute phase) to decompensated values at 20 weeks (**Central Illustration**) has also been observed in patients. Cameli et al. [21] have shown that LAS(r) is increased in patients with mild MR. On the other hand, patients with more severe MR showed a progressive impairment of LAS(r). Debonnaire et al. [5] investigated the correlation between the grade of LA fibrosis and LA function in patients with severe MR referred for mitral surgery. They demonstrated a stepwise reduction of LAS(r) with the lowest values in patients with more severe fibrosis at histologic analysis. Although it seems obvious that fibrosis is the cause of reduced LAS(r), we have shown in our computer simulations that LA eccentric hypertrophy alone can also lead to a reduction of LAS(r) (Fig 5). The reduction in LA myocardial function is therefore likely a result of eccentric hypertrophy in combination with fibrosis.

### Limitations

The in-vivo model used in this study was originally created to investigate the electromechanical effects of LBBB and therefore not a primary MR study. As a result, no typical control group was available as LBBB was present in both canine groups. Therefore, we evaluated the effects of MR, LBBB and the combination on LA structure and function using computer simulations. Both in-vivo experiment as computer simulations suggest that the observed LA dilation and functional deterioration were related to MR rather than LBBB.

Histology and gene expression analysis show great variation. Due to the large variation and low numbers, there may be insufficient statistical power to demonstrate a difference. However, a trend towards interstitial fibrosis formation in the MR-LBBB was demonstrated.

Our experimental data consisted of young and healthy mongrel dogs and do clearly differ from the pathophysiological heterogeneity expected in human patients. The LA was completely normal at baseline, while in clinical practice LA remodeling often has already occurred before onset of MR due to hypertension, atrial fibrillation, other valvular disease and/or heart failure. Our model is more representative of clinical cases for acutely ruptured chordae in patients with pre-existing valve prolapse or during blunt chest trauma. However, the design of the study makes it possible to provide clear insight into the evolution of LA remodeling after acute MR and showed that not only interstitial fibrosis causes decrease in LA function. This study also illustrates that LA dysfunction precedes LV failure.

## Conclusion

In a canine model of acute MR, LA reservoir and contractile function augmented to supranormal values in absence of significant LA dilation. Over time, there is a gradual decrease in both strain values (pseudonormal to decompensated) together with progressive LA dilation. Histology and computer simulations suggest that these functional changes are the result of a combination of LA eccentric hypertrophy and fibrosis. These mechanistic insights in LA pathophysiology may aid in further understanding the effects of LA volume overload in patients with MR and can help to find the optimal timing for surgery.

## Supporting information

S1 Graphical abstract Left atrial remodeling after acute primary mitral regurgitation. (DOCX)

S1 Appendix (DOCX)

S1 Dataset (XLSX)

## Abbreviations

CO: Cardiac output
EROA: Effective regurgitant orifice area
HR: Heart rate
LA: Left atrium/atrial
LAFAC: Left atrial fractional area change
LAS(r): Left atrial reservoir strain
LAS(ct): Left atrial contractile strain
LBBB: Left bundle branch block
LV: Left ventricle/ventricular
LVEDP: Left ventricular end-diastolic pressure
LVEDV: Left ventricular end-diastolic volume
LVEF: Left ventricular ejection fraction
MAP: Mean arterial pressure
MR: Mitral regurgitation
RF: Regurgitant fraction
