# Supplementary material for: Left atrial remodeling in mitral regurgitation: A combined experimental-computational study
Source: PLoS One. 2022 Jul 15;17(7):e0271588. doi: 10.1371/journal.pone.0271588 (PMC9286246; doi:10.1371/journal.pone.0271588)
Supplement: S1 Graphical abstract — (DOCX) [file pone.0271588.s001.docx]

**Graphical Abstract:** Left atrial remodeling after acute primary mitral regurgitation


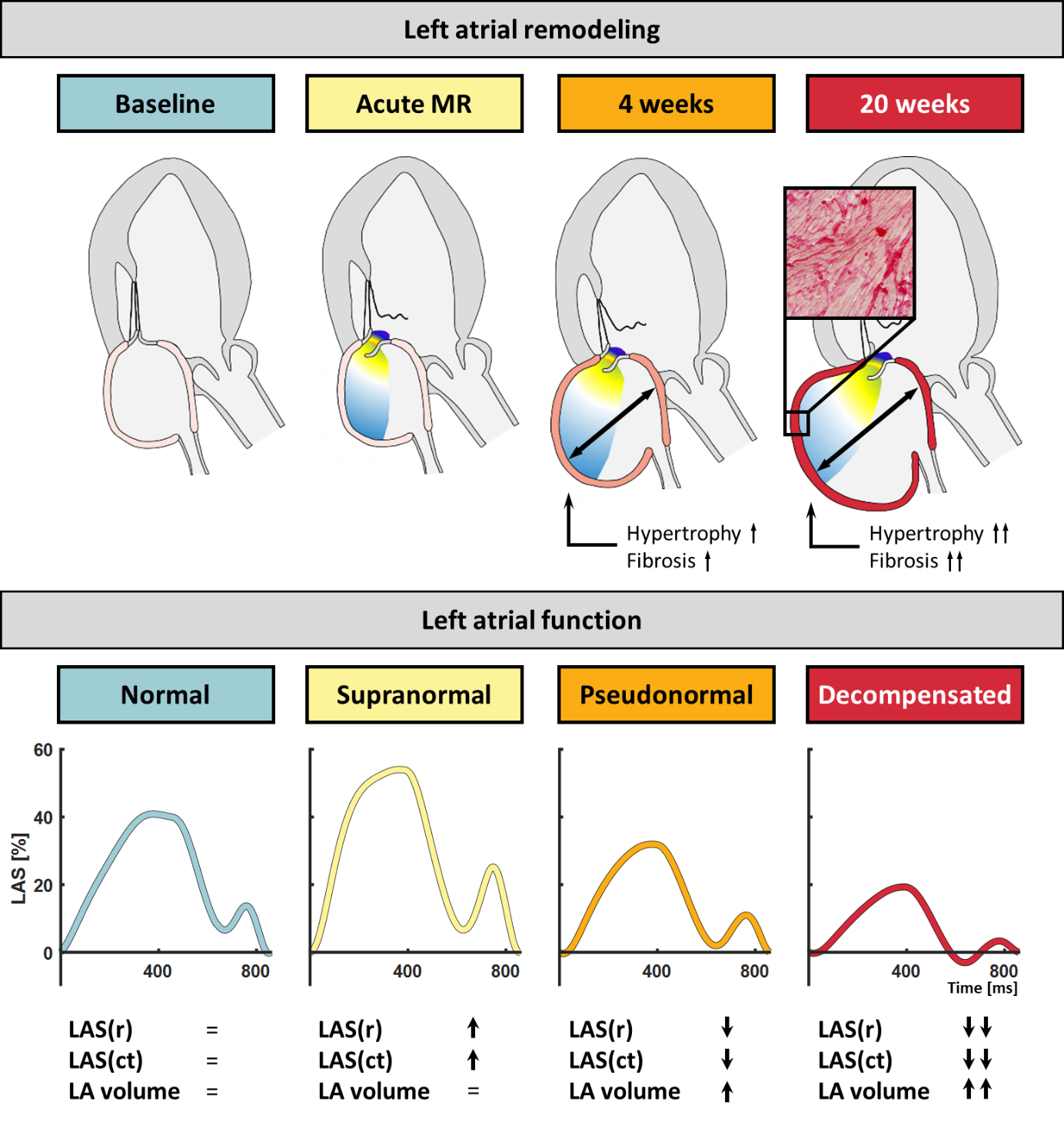


Introduction of acute MR augmented LA reservoir and contractile strain to supranormal values, accompanied by insignificant LA dilation. At 4 and 20 weeks, there is a gradual decrease in both strain values (pseudonormal and decompensated, respectively) with progressive LA dilation, which was correlated to LA eccentric hypertrophy and fibrosis.
