# Supplementary material for: Left atrial remodeling in mitral regurgitation: A combined experimental-computational study
Source: PLoS One. 2022 Jul 15;17(7):e0271588. doi: 10.1371/journal.pone.0271588 (PMC9286246; doi:10.1371/journal.pone.0271588)
Supplement: S1 Appendix — (DOCX) [file pone.0271588.s002.docx]

**Left atrial remodeling in mitral regurgitation: a combined experimental-computational study**

*Bouwmeester & Van Loon et al.*

**Supplemental Material**

# Supplementary appendix 1. Computational modelling

## The CircAdapt Model

The CircAdapt model [1, 2] is a closed-loop lumped parameter model of the adult human heart and circulation that enables realistic simulation of beat-to-beat cardiovascular hemodynamics and mechanics. Cardiac hemodynamics and tissue mechanics are described through a phenomenological model of active and passive myofiber mechanics. Direct ventricular interactions are modelled using the TriSeg module using the concept of conservation of energy [1]. Wall heterogeneities were modelled using the MultiPatch module [2]. The valves are modelled as a narrow orifice whose area varies during a cardiac cycle, described by the Bernoulli equation. Valvular flows in healthy and disease have been validated extensively through direct comparison both with clinical Doppler recordings and with derived echocardiographic parameters [2, 3]. Furthermore, an importance feature of the CircAdapt model is the structural adaptation of cardiac and vascular wall thickness, area, and passive stiffness to normalize tissue loading [5].

### Homeostatic pressure-flow regulation

Circulating blood volume and systemic vascular resistance are altered to maintain mean arterial pressure (MAP) and cardiac output (CO) through homeostatic pressure-flow regulation, representing venous pooling and fluid retention or excretion by the renin-angiotensin-aldosterone system. When homeostatic pressure-flow regulation is enabled in the model, the ratio of the current MAP to the target MAP is calculated after each simulation of a single cardiac beat, as is the ratio of the current CO to the target CO.

## Healthy reference simulation

The default CircAdapt model parametrization by Walmsley et al [2] did not produce qualitatively similar left atrial (LA) strain as compared to the experimental data (**Supplemental Figure 1**), with too low LA reservoir strain and too high LA contractile strain in the simulations. To obtain a healthy reference simulation which resembles to experimental data more, we used a particle swarm optimization (PSO) algorithm with simulated annealing (SA-PSO) as previously described by Yang et al [6] to parameterize the model. The various steps in obtaining this reference simulations are described below.

###
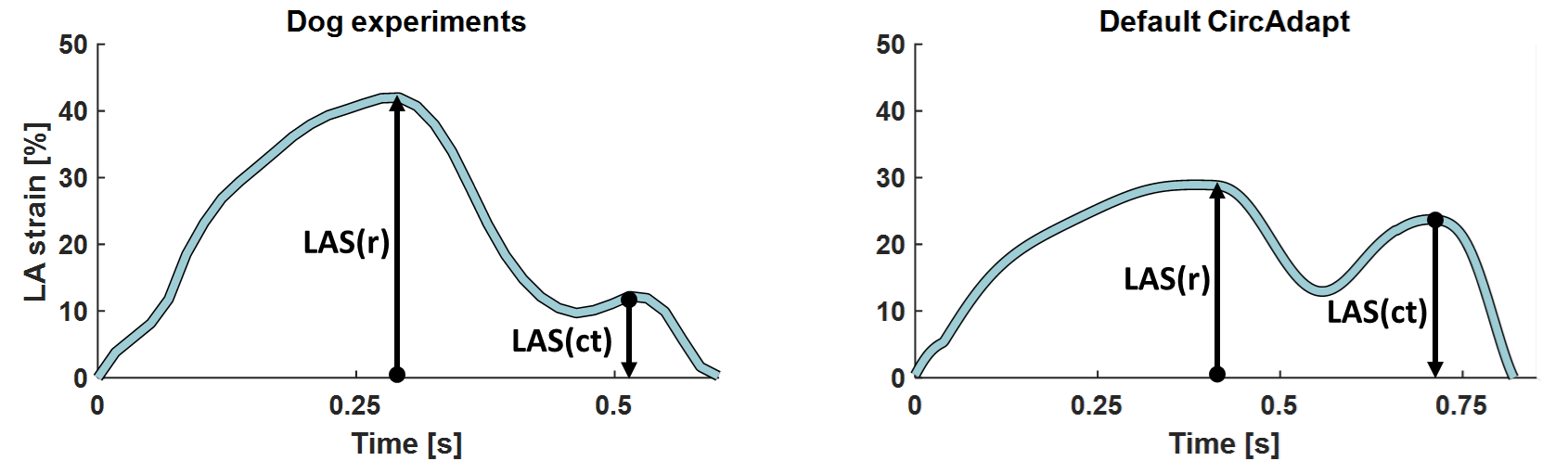


**Supplemental Figure 1.** LA strain comparison between dog experiments and default CircAdapt model parameterization in the baseline phase. Note that the default model parameterization results in too low LA reservoir strain (LAS(r)) and too high LA contractile strain (LAS(ct)).

### Baseline phase

First, the baseline phase (i.e., prior to acute MR) was simulated by a mitral valve effective regurgitant orifice area (EROA) of zero. Using the abovementioned homeostatic pressure-flow regulation, CO and MAP were set to 5.1 L/min and 92 mmHg. Heart rate was fixed at 70 beats/min. When hemodynamic stability was reached LA strain and LA strain rate were calculated and compared to the experimental data at baseline. In addition, simulated maximum LA volume (LAV), LV end diastolic volume (LVEDV), LV ejection fraction (LVEF) and LV global longitudinal strain (LV GLS) were compared to healthy reference values from pooled data on typical normal cardiac function [7].

### Acute MR phase

To ensure that simulating an acute severe MR would acutely change LA strain similar to the dog experiments, information on LA strain from the acute MR phase was included in the optimization algorithm. The acute MR phase was simulated by an increase EROA to 0.40 cm^2^, and by a reduced CO of 3.6 L/min and MAP of 75 mmHg, to represent the compromised hemodynamics in MR [8]. Homeostatic pressure-flow regulation was enabled. All other model parameters were unchanged as compared to the baseline simulation. When hemodynamic stability was reached, LA strain and LA strain rate were calculated and compared to the experimental data acutely after MR.

### Cost function

The optimization algorithm aims to minimize the cost function, which is defined as the sum squared difference between measurements and model outputs. The cost function includes 1) LA strain at baseline and after MR, 2) LA strain rate (first-order time-derivative) at baseline and after MR, 3) maximum LA volume (LAV), 4) LV end-diastolic volume (EDV), 5) LV ejection fraction (EF), 6) and LV global longitudinal strain (GLS). These indices were selected so that LA strain would resemble the observations of the dog experiments at baseline and acutely after MR as well as to ensure normal LA and LV function.

### Model parameters

An important aspect of CircAdapt model is that material parameters can be automatically adapted to obtain physiological behavior of heart and vessel walls [5]. Material parameters governed by adaptation include cardiac and vascular wall thickness, area, and stiffness. This adaptation module was used to the purpose of creating a healthy reference simulation that better resembles the dog experiments. Model parameters to optimize included all 8 adaptation parameters, as well as 12 parameters belonging to cardiac contractile behavior and 2 vascular function parameters.

### Optimization algorithm

A brief description of the optimization algorithm will be provided here; for further methodological details we refer to the study by Yang et al. [6]. SA-PSO was used to minimize the abovementioned cost function using the model parameters so that LA strain at baseline and acutely after MR resembles the dog experiments. A PSO algorithm is a broadly used stochastic parameter optimization algorithm that is highly suitable for non-linear optimization problems [9]. In previous studies, PSO has been shown to successfully parameterize the CircAdapt model [10, 11]. However, PSO has the potential to convergence prematurely [12]. To address this shortcoming, adaptations have been made such as combining PSO with simulated annealing algorithm. SA-PSO has been shown to improve the global search ability by accepting non-optimal particle solutions given a probability (referred to as ‘temperature’) in the early phase of the optimization protocol [6, 13, 14]. In SA-PSO, a particle’s trajectory is more erratic at high temperature (i.e., high probability of a proposed particle solution being accepted) compared to low temperature thereby exploring more of the model parameter space. After each iteration, the probability of a proposed particle solution being accepted is reduced (referred to as ‘cooling’) [6].

The optimization algorithm was initiated by performing 5000 quasi-random Monte Carlo (MC) simulations. For each MC simulation, the cost function was calculated from which a total of 20 initial candidate solutions were selected with the lowest cost function value. The initial temperature was set equal to the highest cost function value of the 20 candidate solutions and the cooling coefficient is set to 0.95. SA-PSO was stopped when the cost function value reduced less than 1% within one iteration. The optimized healthy reference simulation is shown in **Supplemental Figure 2**.

**Supplemental Figure 2.** Healthy reference simulation with LA strain function similar to the dog experiments and normal LV function.

# Supplemental appendix 2. Left atrial substrate simulations

To determine the potential mechanism underlying the changes in LA function following acute MR phase, LA eccentric hypertrophy and passive stiffness substrates, which mimic the expected (patho-)physiological changes, were simulated.

- **LA eccentric hypertrophy** is a compensatory remodeling response to volume overload, in which the cavity dilates, and myocardial wall thickness remains constant. As a result, the operative sarcomere length remains unchanged in eccentric hypertrophy. In CircAdapt, eccentric hypertrophy is simulated by increasing wall reference area from 100% to 300% of its reference value, while maintaining wall thickness.
- **LA passive stiffness** is the stress generated by the passive deformation of the soft tissue making up the myocardium. To simulate increased LA passive stiffness (i.e., reduced compliance), the scaling factor of passive stiffness of the entire LA myocardium was increased from 100% (normal passive stiffness) to maximum of 600% of its normal value.

Each substrates specific ranges were obtained until either the change in LA dilation (i.e., LA end-systolic area) or function (i.e., LA reservoir and contractile strain) of the 20 weeks phase from experimental data were reproduced.

# Supplemental appendix 3. Best-match simulations

Three LA functional indices that were used to determine the best-match simulation to the experimental data of the 4 and 20 weeks phases after MR, namely LAS(r) ($x$), LAS(ct) ($y$) and LAESA ($z$). For each simulation, we varied the LA eccentric hypertrophy and LA passive stiffness severity and compared them with the average value of each respective phase. The simulation with the lowest $Relative Error$ compared to the average LAS(r), LAS(ct) and LA end-systolic area values in the 4 and 20 weeks phases after MR were selected as the best-match simulation. $Relative Error$ was defined as:

$Relative Error\left( i \right) = \frac{\left| x_{sim}-x_{exp}\left( i \right) \right|}{std\left( x_{exp}\left( i \right) \right)}+\frac{\left| y_{sim}-y_{exp}\left( i \right) \right|}{std\left( y_{exp}\left( i \right) \right)}+\frac{\left| z_{sim}-z_{exp}\left( i \right) \right|}{std\left( z_{exp}\left( i \right) \right)}$,

where $sim$ indicates simulated index values, $exp$ is used for average index values measured in the animal experiments per phase (indicated by $i$). The absolute difference between $sim$ and $exp$ of an index is divided by its standard deviation per phase as normalization factor. **Supplemental Figure 3** shows the $Relative Error$, LAS(r), LAS(ct) and LA end-systolic area for all simulation to the 4 and 20 weeks phase after MR. The best-match simulation for each phase is indicated by the white circle.


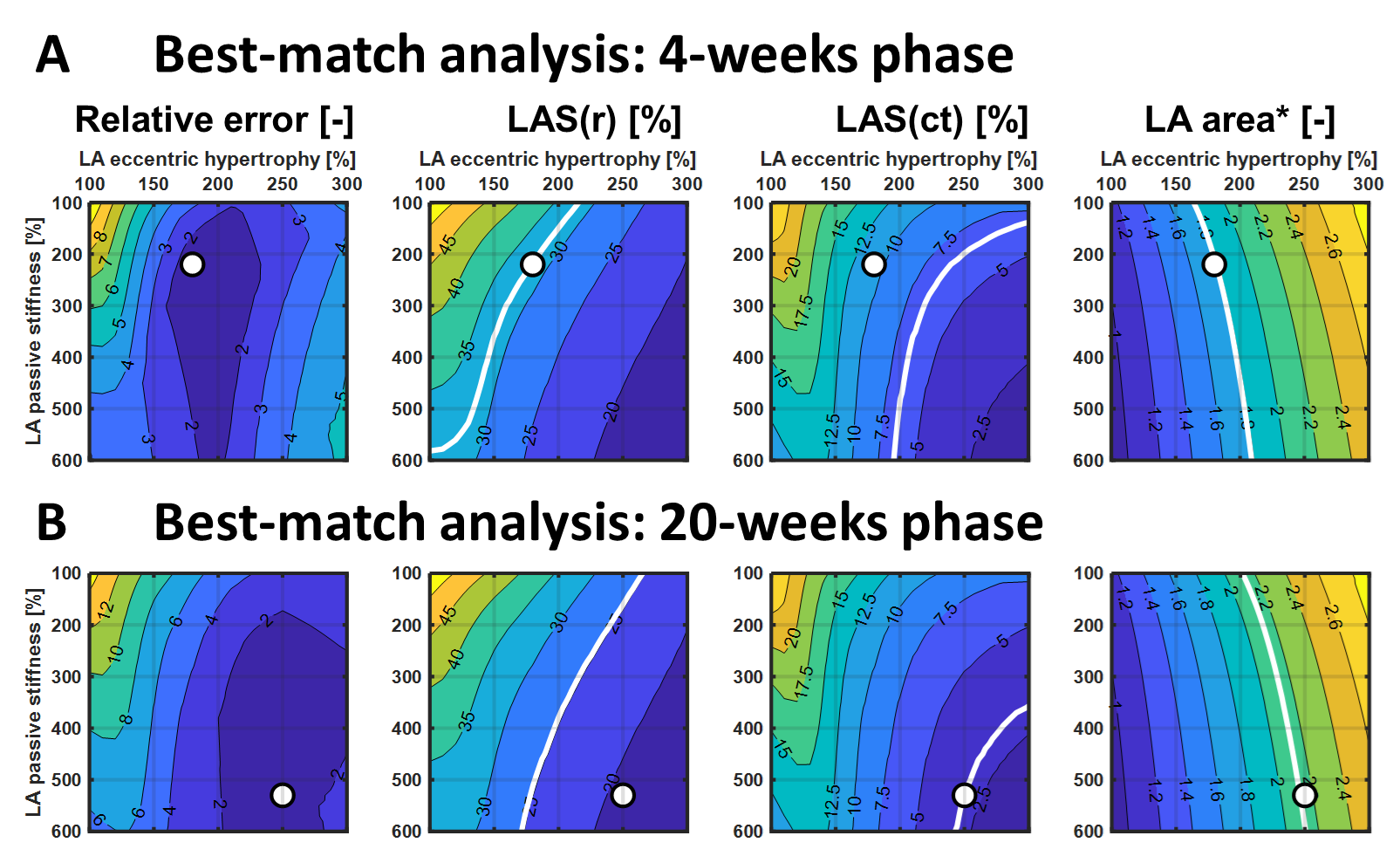


**Supplemental Figure 3.** Best-match analysis based on the minimum sum squared error (relative error), as well as the simulated effects on LAS(r), LAS(ct) and LA end-systolic area relative to baseline (LA area*). The best-match simulation for each respective phase is indicated by the white circle. The white isolines in the LAS(r), LAS(ct) and LAA maps indicate the experimental measurement in the 4 and 20 weeks phase, respectively.

# References

| [1] | Lumens J, Delhaas T, Kirn B, et al. Three-wall segment (TriSeg) model describing mechanics and hemodynamics of ventricular interaction. Ann Biomed Eng. 2009;37(11):2234–55. |
| --- | --- |
| [2] | Walmsley J, Arts T, Derval N, et al. Fast Simulation of Mechanical Heterogeneity in the Electrically Asynchronous Heart Using the MultiPatch Module. PLoS Comput Biol. 2015;11(7):1–23. |
| [3] | Palau-Caballero, G., Walmsley, J., Gorcsan III, J., Lumens, J., & Delhaas, T. (2016). Abnormal ventricular and aortic wall properties can cause inconsistencies in grading aortic regurgitation severity: a computer simulation study. *Journal of the American Society of Echocardiography*, *29*(11), 1122-1130. |
| [5] | Arts, T., Lumens, J., Kroon, W., & Delhaas, T. (2012). Control of whole heart geometry by intramyocardial mechano-feedback: a model study. *PLoS Comput Biol*, *8*(2), e1002369. |
| [6] | Yang X., Niu J., & Cai Z. (2018). Chaotic simulated annealing particle swarm optimization algorithm. IMCEC 2018. IEEE |
| [7] | Kawel-Boehm N, Hetzel SJ, Ambale-Venkatesh B et al. Reference ranges ("normal values") for cardiovascular magnetic resonance (CMR) in adults and children: 2020 update. J Cardiovasc Magn Reson 2020;22:87. |
| [8] | Walmsley, J., Squara, P., Wolfhard, U., Cornelussen, R., & Lumens, J. (2019). Impact of abrupt versus gradual correction of mitral and tricuspid regurgitation: a modelling study. *EuroIntervention: journal of EuroPCR in collaboration with the Working Group on Interventional Cardiology of the European Society of Cardiology*, *15*(10), 902-911. |
| [9] | Eberhart, R., & Kennedy, J. (1995, November). Particle swarm optimization. In *Proceedings of the IEEE international conference on neural networks* (Vol. 4, pp. 1942-1948). |
| [10] | van Osta, N., Lyon, A., Kirkels, F., Koopsen, T., van Loon, T., Cramer, M. J., ... & Lumens, J. (2020). Parameter subset reduction for patient-specific modelling of arrhythmogenic cardiomyopathy-related mutation carriers in the CircAdapt model. *Philosophical Transactions of the Royal Society A*, *378*(2173), 20190347. |
| [11] | Koopsen, T., van Osta, N., van Loon, T., … & Lumens, J. (2022). A lumped two-compartment model for simulation of ventricular pump and tissue mechanics in ischemic heart disease. *Front. Physiol., 13: 782592*. |
| [12] | Clerc, M., & Kennedy, J. (2002). The particle swarm-explosion, stability, and convergence in a multidimensional complex space. *IEEE transactions on Evolutionary Computation*, *6*(1), 58-73. |
| [13] | Sudibyo, S., Murat, M. N., & Aziz, N. (2015, May). Simulated annealing-Particle Swarm Optimization (SA-PSO): Particle distribution study and application in Neural Wiener-based NMPC. In *2015 10th Asian Control Conference (ASCC)* (pp. 1-6). IEEE. |
| [14] | Gao, W. F., Liu, S. Y., & Huang, L. L. (2012). Particle swarm optimization with chaotic opposition-based population initialization and stochastic search technique. *Communications in Nonlinear Science and Numerical Simulation*, *17*(11), 4316-4327. |
